# Ant nest architecture is shaped by local adaptation and plastic response to temperature

**DOI:** 10.1038/s41598-021-02491-w

**Published:** 2021-11-29

**Authors:** Madison Sankovitz, Jessica Purcell

**Affiliations:** Department of Entomology, University of California, Riverside, 900 University Ave., Riverside, CA 92521 USA

**Keywords:** Evolutionary ecology, Experimental evolution

## Abstract

Social insects are among the most abundant arthropods in terrestrial ecosystems, where they provide ecosystem services. The effect of subterranean activity of ants on soil is well-studied, yet little is known about nest architecture due to the difficulty of observing belowground patterns. Furthermore, many species’ ranges span environmental gradients, and their nest architecture is likely shaped by the climatic and landscape features of their specific habitats. We investigated the effects of two temperature treatments on the shape and size of nests built by *Formica podzolica* ants collected from high and low elevations in the Colorado Rocky Mountains in a full factorial experiment. Ants nested in experimental chambers with soil surface temperatures matching the local temperatures of sample sites. We observed a plastic response of nest architecture to conditions experienced during excavation; workers experiencing a high temperature excavated deeper nests than those experiencing a cooler temperature. Further, we found evidence of local adaptation to temperature, with a significant interaction effect of natal elevation and temperature treatment on nest size and complexity. Specifically, workers from high elevation sites built larger nests with more tunnels when placed in the cool surface temperature treatment, and workers from low elevation sites exhibited the opposite pattern. Our results suggest that subterranean ant nest architecture is shaped by a combination of plastic and locally adapted building behaviors; we suggest that the flexibility of this ‘extended phenotype’ likely contributes to the widespread success of ants.

## Introduction

The nests of social insects have doubtlessly contributed to their widespread ecological success by providing shelter and defensive benefits^1,2^. Ants (Formicidae) exhibit particularly diverse nesting strategies, which include nesting in soil, leaf litter, plants, and cavities, allowing them to persist in most terrestrial habitats. Many ant species nest in soil, modifying soil ecosystems in the process^3^. They increase soil drainage and aeration by forming underground tunnels and chambers and incorporate nutrients into soil through food storage and the accumulation of feces and corpses^4^. Hence, the benefits of ant nests are multifaceted, providing advantages to both ecosystem functioning and colony fitness.

Numerous studies measured above-ground aspects of ant nests^5,6^, but subterranean nest architecture has received comparatively little research attention, likely due to the difficulties of observing and measuring this belowground aspect of ant life (but see^7,8^). The structure, or architecture, of ant nests allows for the precise regulation of some environmental conditions. For example, large colonies of wood ants (*Formica* rufa group) build elaborate and long-lasting thatch mounds constructed of plant materials and mineral soil, reducing moisture loss and regulating temperature for optimal colony growth and performance^9,10^. Temperature is an important aspect of colony growth and survival^11^; the centers of these nests are particularly important for providing a favorable microclimate for brood development^12^. Nest depth predominantly affects microclimatic conditions faced by colonies with subterranean nests^13^ since temperature, humidity, and air composition vary predictably with soil depth^14^. Yellow meadow ants (*Lasius flavus*) respond to taller vegetation by building larger mounds with soil excavated from deeper soil layers, thereby changing the shape of the mound to optimize the collection of solar radiation^15^. Harvester ants (*Pogonomyrmex* spp.) create and maintain vegetation-free zones around their nests by removing debris and clipping the vegetation, reducing transit time for foragers, decreasing fire and predation risk, and increasing exposure to solar radiation^16^. These examples show that nest construction can lead to decreased environmental hazards and enhance conditions for colony development. As Jones et al.^17^ postulated, ants’ nest structures are intentional responses to their environmental surroundings and thus an inferred extended plastic phenotypic trait that may allow ants to occupy many different habitats. Therefore, as colonies’ needs vary with time and environment, the properties of nests likely also change in response^18^.

In addition to providing environmental stability, ant nest architecture shapes and, in turn, is shaped by collective behavior and therefore provides an opportunity to study individual- and colony-level behaviors in a shared, dynamic environment^8^. Physiology and individual-level behavioral variation can have colony-level effects reflected in nest architecture; for example, a building pheromone added by individual workers to the nest material has been shown to be a critical factor that controls the growth and form of nest architecture^19^. Similarly, in yellow meadow ant colonies, the angular distribution of tunnels is probably a result of local competition among workers^1^. Likewise, nest architecture can influence colony-level behavior; as harvester ant nest chamber connectivity and redundancy of connections among chambers increase, so does a colony’s speed of recruitment to food^8^.

Most previous studies on subterranean nest architecture have been conducted in a single habitat, yet many ant species ranges span distinct habitats and climates, some of which are rapidly changing with climate and other anthropogenic disturbances^20^. Global warming has stimulated worldwide studies aiming to assess or predict the impact of rising environmental temperatures on organisms^21–23^. Many of these studies have focused on thermal tolerances of terrestrial ectotherms^24–26^ because they represent the vast majority of terrestrial biodiversity^27^ and are especially likely to be vulnerable to climate warming due to the strong influence of environmental temperature on their physiological and behavioral functions^28^. Social insects, including ants, provide a unique opportunity to study how behaviors could mitigate the impact of warming, as temperature affects the performance of both individuals and the colony as a whole. Since nest architecture affects and reflects the thermoregulation ability of a colony, it likely plays a vital role in the ability of these insects to respond to changing climate conditions.

*Formica podzolica* (Francoeur, 1973) is a montane ant with colonies ranging from 5000 to 100,000 workers. They construct conspicuous soil mound nests, which can exceed 2 m in diameter^29^. Nests occur in pine and aspen stands from Alaska to New Mexico, at altitudes up to approximately 3000 m, so this species is ideal for studying how local adaptation and extrinsic conditions shape nest architecture. Although no published descriptions of complete nests exist to our knowledge, alteration of nest architecture may be a key to these ants’ survival in a wide range of environments. In this study, we investigate the extent to which the extended phenotype of nest architecture is either (i) plastic and varies with soil surface temperature or (ii) locally adapted to the population’s native climate. Using custom-built nest boxes and temperature chambers, we carried out a laboratory transplant experiment with populations of *F. podzolica* from two different elevations separated by ~ 1000 m. We measured nest size (depth and area) and complexity (number of tunnels) daily during week-long trials.

## Materials and methods

**Figure 1:**
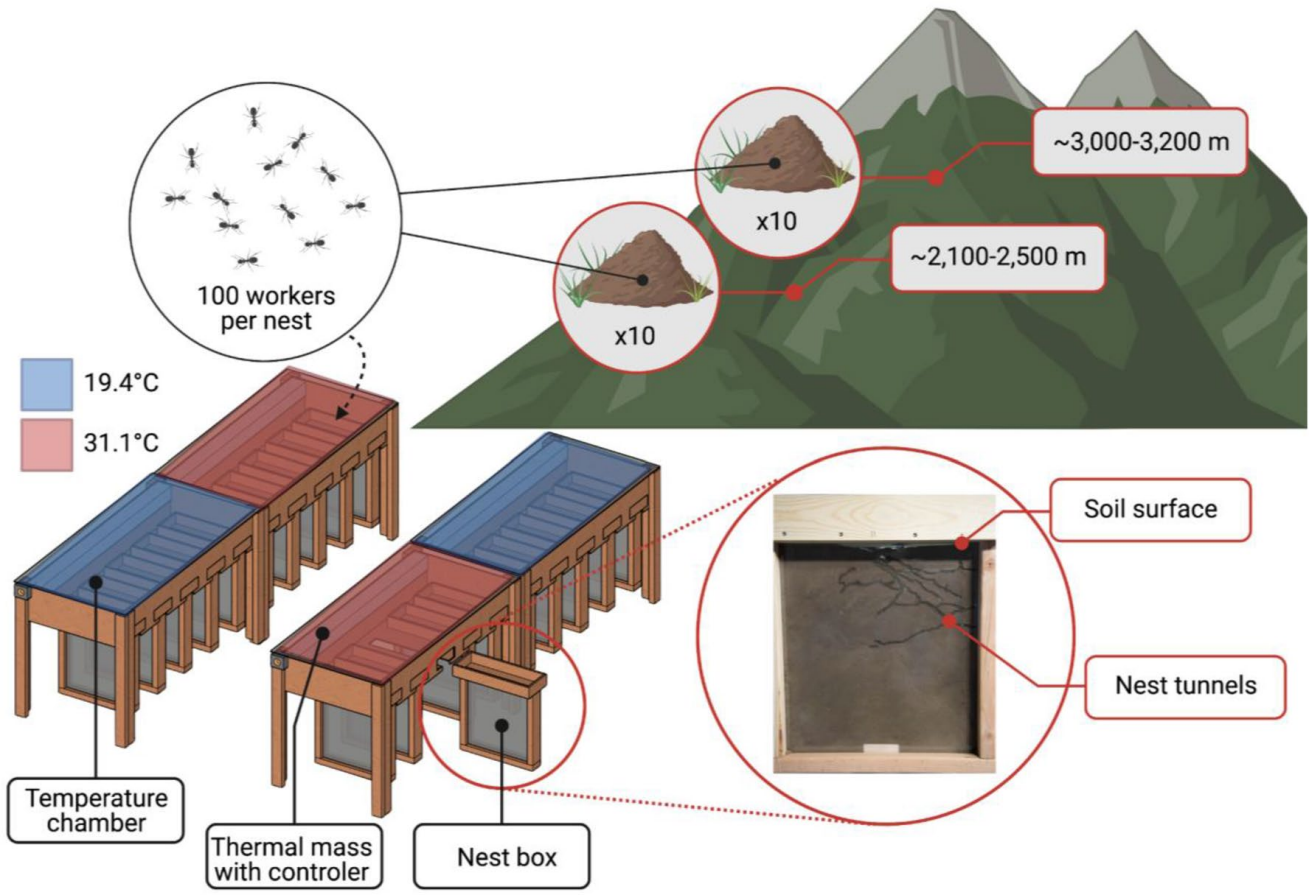
Overview of experimental design. We collected 100 workers from each of 20 colonies per trial (10 from higher elevation sites and 10 from lower elevation sites). We allowed colonies to excavate nests in boxes attached to temperature chambers at the soil surface level. There were four chambers, each containing five nest boxes and a mixture of low- and high-elevation colonies. We repeated this experiment three times during one summer to obtain a total sample of 60 colonies. Created with BioRender.com.

We compared nests excavated by *F. podzolica* workers (collected from a total of 60 mature colonies) from sites within two elevational ranges under two temperature treatments (Fig. 1). We collected colonies in Boulder County, CO, USA, during June–September of 2019. Lower elevation sites spanned ~ 400 m and included Platt-Rogers Memorial Park (39.98′ N, 105.44′ W, ~ 2100 m), Mud Lake Open Space (39.98′ N, 105.51′ W, ~ 2500 m), and Reynolds Ranch (40.17′ N, 122.24′ W, ~ 2200 m). Higher elevation sites spanned ~ 200 m and included areas surrounding the Sourdough Trail (40.02′ N 105.31′ W, ~ 3000 m) and Brainard Lake Recreation Area (40.08′ N, 105.57’ W, ~ 3200 m). Although landscape features are similar across all sites (closed-canopy pine forest with limited undergrowth diversity), daily temperatures vary significantly (see below).

During each of three 1-week trials, we collected 100 workers directly from the nest entrance of each of 20 colonies (10 from low elevation sites and 10 from high elevation sites each time) using forceps. Overall, we collected 20 colonies per trial, for a total of 60 colonies. We sampled nests across all sites for each trial, and colonies were not re-sampled during the experiment. We housed the workers in 20 transparent nest boxes (45.72 × 50.8 × 13.81 cm) in a laboratory setting and allowed them one week to excavate nests. Boxes contained a mixture of soil in equal parts from each site that we sieved (0.635 cm), mixed thoroughly, and compacted at a constant rate across boxes. The wood boxes had glass walls and were kept dark with black fabric to mimic a subterranean habitat. Four temperature chambers (also constructed of wood) encapsulated five boxes each from the soil surface up, with the majority of each nest box extending below the chambers and in contact with the ambient air temperature of the room. This setup mimics the expected natural temperature gradient, with the soil surface warm during the day and the deeper parts of the nest cooler. Temperature chambers were heated via a thermal mass consisting of a concrete mortar mix surrounding radiant heating tubing connected to a temperature-controlling outlet. We monitored the soil temperature of one box per chamber per trial using iButton temperature sensors at the surface, halfway deep, and bottom of the boxes (Fig. S1).

We offered ants plain water and sugar water in cotton-plugged Olympus 1.7 ml Microtubes, placed at the soil surface inside each box. To test the effects of temperature on nest architecture, we applied a high-temperature treatment to 10 boxes (31.1 °C, the July high temperature averaged from 1981 to 2010 in Nederland, CO at ~ 2400 m; National Oceanic and Atmospheric Administration (NOAA) 2019) and a cooler temperature treatment to the other 10 boxes (19.4 °C, the July high temperature averaged from 1981 to 2010 in Ward, CO at ~ 2850 m; NOAA 2019). These temperature treatments were taken from NOAA Weather Observation Stations near our low- and high-elevation sites. The ambient air temperature in the room that contained the boxes was kept at 15.5 °C. We exposed half of the low-elevation colony fragments to the high-temperature treatment at the soil surface and the other half to the low-temperature treatment at the soil surface, with the same treatments for the high-elevation colony fragments. We replicated the experiment three times for a total of 60 colonies.

**Figure 2:**
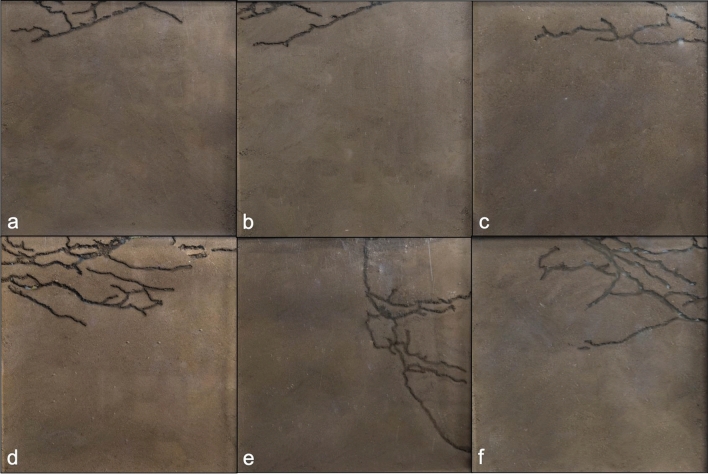
Examples of nests after the 7-day excavation period. (**a**–**c**) are nests from colonies that experienced the low-temperature treatment, whereas (**d**–**f**) are nests from colonies that experienced the high-temperature treatment.

Every day throughout each trial, we took photographs of both sides of every box to measure the following attributes of nest architecture: nest depth, area of soil excavated (measured as the combined tunnel area from both sides of the nest box), and number of tunnels. We defined a tunnel as one branch of uninterrupted excavated soil between nodes, connection points between two or more tunnels. We imported digital photographs into *ImageJ*, calibrated a 0.0121 cm/pixel scale, and took measurements by tracing the length of each nest, counting the number of pixels making up the tunnels in the photographs (using the high contrast area measurement tool), and counting the number of tunnels^30^. Nests varied greatly in their shape, ranging from one simple tunnel to complex networks of tunnels (Fig. 2). Nine colony fragments did not excavate nests during the experiment; these were spread out across the four different treatments and excluded from all analyses (Table S2).

To determine if the natal environment and temperature treatment influenced nest size and complexity, we compared nests excavated by colony fragments from two natal elevations. In our analysis we used two types of models. First, we used two-way analysis of variance (ANOVA) models with a random effect (R v. 5.3.5, package *stats*^31^) to compare the nest architecture metrics between high- and low-elevation colony fragments tested in high and low temperatures after one week of building. Nest depth, area of soil excavated, and number of tunnels at the end of each one-week trial represented our response variables, colony natal elevation (low or high), temperature treatment, and their interaction were fixed effects, and colony collection site was a random effect. We confirmed the homogeneity of variance using a Levene's test from the *car* package^32^.

Second and in complement we used a repeated-measures ANOVA (*stats* package) to compare nest architecture measurements across all seven days of observation to assess whether excavation progressed at the same rate between treatments. We removed observations with a value of 0 (some colony fragments took a day or two to begin excavation). Nest depth, area of soil excavated, and number of tunnels on each day represented our response variables, colony natal elevation, temperature treatment, day, and their interaction were fixed effects, and colony collection site was a random effect. To determine at what day nest depth began to differ between temperature treatments, we compared estimated marginal means of depth per temperature treatment between each day (*emmeans* package) with a Bonferroni correction for multiple comparisons.

## Results

**Figure 3:**
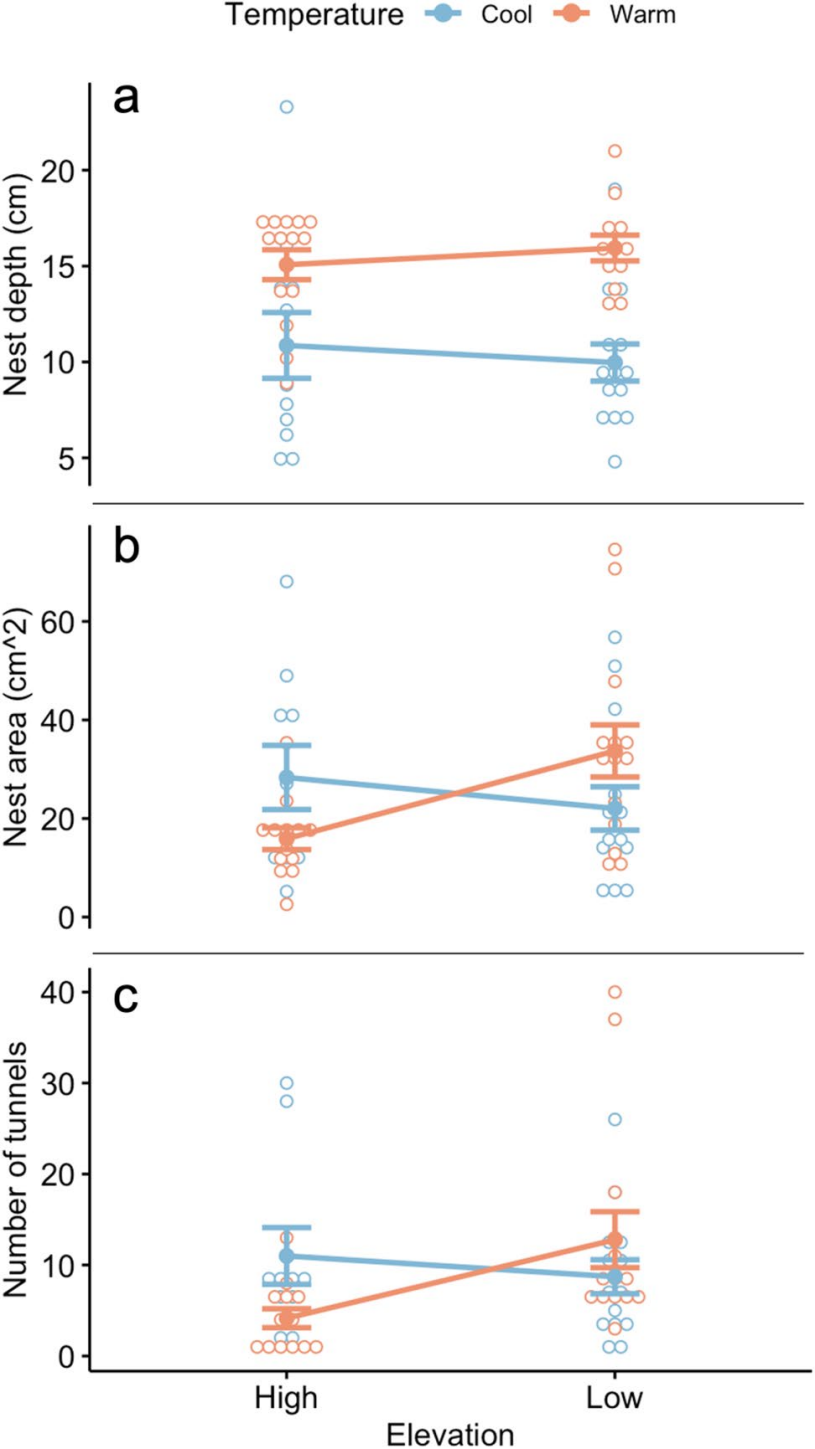
Nest depth (**a**), area of soil excavated (**b**), and the number of tunnels (**c**) excavated by low- and high-elevation workers under cool (blue) and warm (red) temperature treatments. Each dot represents one observation, and error bars show standard error. Workers experiencing the warm treatment excavated significantly deeper nests than those under the cool treatment, while the tunnel area and the number of tunnels depended on the interaction between the workers’ native elevation and the temperature treatment.

Over the seven days of observation, groups of 100 workers produced nests ranging from 4.7 to 23.3 cm in total depth. Nest depth differed significantly between temperature treatments (Table 1, Fig. 3a). The nests excavated by workers experiencing the warmer soil surface temperature treatment (31.1 °C) were, on average, 1.5 times deeper (15.5 cm, s.e. 0.5) than those produced by workers experiencing the cooler temperature treatment (19.4 °C, 10.4 cm, s.e. 0.91).

**Table 1:** Two-way ANOVA results for Day 7 (fully excavated) nests.

Response variable	Independent variable	F	P
Nest depth (cm)	Elevation	0.220	0.642
	Temperature	23.353	1.48e-05*
	Elevation × temperature	0.694	0.409
Tunnel area (cm^2^)	Elevation	1.943	0.1699
	Temperature	0.037	0.8476
	Elevation × temperature	6.473	0.0143*
Number of tunnels	Elevation	2.289	0.1370
	Temperature	0.114	0.7375
	Elevation × temperature	5.157	0.0278*

Nest depth differed significantly between temperature treatments. There was a significant interaction effect between temperature treatment and natal elevation for both tunnel area and the number of tunnels. Df = 1,47.

*Significant, ⍺ = 0.05.

The area of the soil excavated ranged between 2.6 and 74.6 cm^2^ and did not differ significantly among temperature treatments or natal elevation (Table 1; Fig. 3b). However, there was a significant interaction effect between temperature treatment and natal elevation (Table 1). Workers from low elevations excavated tunnels with 2.1 times more area (33.7 cm^2^, s.e. 5.3) than those from high elevations (15.9 cm^2^, s.e. 2.2) while experiencing the warm temperature treatment. Conversely, workers from high elevations excavated tunnels with 1.5 times more area (28.3 cm^2^, s.e. 6.5) than those from low elevations while experiencing the cool temperature treatment (22.0 cm^2^, s.e. 4.4).

Nests contained between 1 and 31 tunnels. The effect of temperature treatment on the number of tunnels was not statistically significant, nor was the effect of natal elevation (Table 1; Fig. 3c). However, there was a significant interaction effect (Table 1). On average, nests built by workers from low elevations had 3.1 times more tunnels (12.8 tunnels, s.e. 3.1) than those produced by workers from high elevations while experiencing the warm temperature treatment (4.2 tunnels, s.e. 1.0). Conversely, nests built by workers from high elevations had, on average, 1.3 times more tunnels (11 tunnels, s.e. 3.1) than those built by workers from low elevations while experiencing the cool temperature treatment (8.7 tunnels, s.e. 1.9). These results provide further evidence that colonies’ response to soil surface air temperature is dependent on their natal environments. There was no standard branching pattern across treatments and depths; instead, tunnel arrangements and individual tunnel length and angle appeared highly variable across colonies.

**Figure 4:**
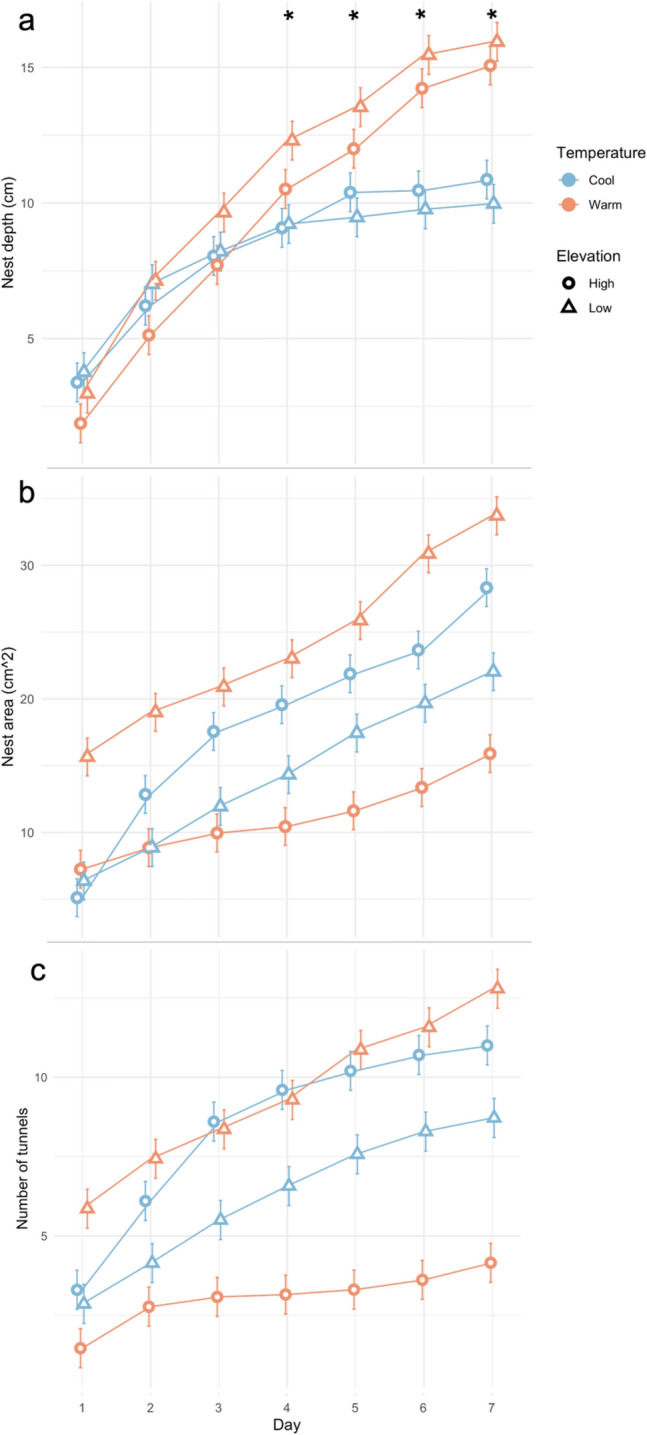
Excavation progress in terms of mean nest depth (**a**), total area (**b**), and the number of tunnels (**c**) throughout the 7-day trials. Error bars represent standard error. *Significant difference between temperature treatments.

Excavation did not progress at the same rate across all seven days of the experiment (Fig. 4). The ants almost universally followed a pattern of making much progress on nest depth at the beginning of the experiment and then decreasing the rate of depth change as they excavated with each subsequent day (Table S1). Conversely, nest area and number of tunnels increased relatively steadily throughout the experiment, although at different rates depending on the colony’s native elevation and temperature treatment. The repeated measures ANOVA revealed that, for nest depth, the interaction of day and temperature is significant, indicating that nests are getting deeper over time but at different rates depending on temperature treatment (Table 2). Both nest area and the number of tunnels increased over time, but the rate did not differ significantly depending on natal elevation or treatment. Nest depth began to significantly differ between temperature treatments on Day 4, and this continued through Day 7 (Table 3).

**Table 2:** Repeated measures ANOVA results for all seven days of the experiment.

Response variable	Independent variable	F	P
Nest depth (cm) df = 6,264	Day	70.448	< 2e-16*
	Day × elevation	1.487	0.183
	Day × temperature	9.046	5.33e-09*
	Day × elevation × temperature	0.086	0.998
Tunnel area (cm^2^) df = 6,272	Day	27.378	< 2e-16*
	Day × elevation	0.618	0.716
	Day × temperature	0.856	0.528
	Day × elevation × temperature	1.865	0.087
Number of tunnels df = 6,272	Day	14.766	1.95e-15*
	Day × elevation	0.757	0.604
	Day × temperature	0.320	0.927
	Day × elevation × temperature	1.232	0.288

The interaction of day and temperature is significant for nest depth.

*Significant, ⍺ = 0.05.

**Table 3:** Estimated marginal means, comparing nest depth under cool and warm temperature treatments.

Day	Estimate	SE	Df	T-ratio	P
1	1.176	1.43	95.7	0.820	0.4141
2	0.233	1.33	73.5	0.175	0.8616
3	1.266	1.33	73.5	0.950	0.3452
4	2.966	1.33	73.5	2.225	0.0291*
5	3.546	1.33	73.5	2.660	0.0096*
6	5.440	1.33	73.5	4.081	0.0001*
7	5.799	1.33	73.5	4.351	< 0.0001*

Nest depth began to significantly differ between temperature treatments on Day 4. Results are averaged over natal elevation.

*Significant, ⍺ = 0.05.

## Discussion

In this study, we observed both a plastic response of nest architecture to conditions experienced during nest construction and evidence of local adaptation to differing conditions in *F. podzolica*. Overall, workers experiencing a high soil surface temperature excavated deeper nests than those experiencing a cooler surface temperature. Neither natal elevation nor temperature treatment alone had a significant effect on the area of soil excavated or the number of tunnels. However, groups collected from lower elevation built larger nests with more tunnels when experiencing warm surface temperatures, while those collected from higher elevation built larger nests with more tunnels when experiencing cool surface temperatures, suggesting that they perform best in conditions more closely matching their natal habitat.

These results add to a growing collection of evidence that abiotic factors such as weather, soil temperature, moisture^33^, and the presence of planes between layers of sediment (ants may excavate horizontal tunnels along such planes)^1^ affect nest structure, an aspect of the extended phenotype of the colony that continually changes as the colony grows. Ant species ranges can span large elevational and latitudinal gradients, and many species are perennial. Owing to the landscape-scale climatic differences across a species’ range, nests built in different localities may differ markedly in form. Although there is evidence of a correlation between soil surface temperature and ambient temperature, respiration, and metabolic rates, further study is needed to determine why soil surface temperature influences nest-building behaviors in *F. podzolica*^34–36^. However, one possibility is that nest depth is a response based on the colony’s thermoregulatory needs. Inhabiting deeper nests may allow for the avoidance of higher temperatures at the surface^37^. This explanation aligns with our observation that the rate of nest depth excavation decreased over the week of the experiment. Ants appeared to eventually settle on a suitable nest depth and focus on excavating larger and more complex nests. Colony fragments experiencing the cool temperature treatment reached this equilibrium faster (around Day 4) than those experiencing the warm temperature treatment, which did not appear to reach an equilibrium. Reducing exposure to surface temperatures by excavating a deeper nest could increase colony longevity because brood development may be optimized at species-specific temperature preferences^38,39^, although the ideal temperature for brood development remains unknown for *F. podzolica*^37^. Conversely, for colonies in cool habitats, building shallow nests may increase the temperature inside the nest chambers toward the species optimum.

Other variables such as soil moisture are also expected to influence the determination of nest depth. Nest depth in the leaf-cutter ant *Acromyrmex landolti*, as an example, has been shown to be negatively correlated with soil moisture^40^, and colonies are known to move fungus gardens vertically through the soil profile in search of more optimal soil moisture. However, soil temperature (both surface and subterranean) generally seems to be a more powerful predictor of nesting habits. For instance, *Acromyrmex crassispinus* colonies build deep subterranean nests in the hot soils of Paraguay, yet superficial ones in the colder thermic soils of central Argentina and southern Brazil^37,41^, although both regions present the same soil moisture regime. We are confident that soil moisture was not a factor driving our results because we standardized it in our experiment. Plasticity in nesting habits based on temperature-sensitive digging may have contributed to the colonization of different habitats worldwide^42^. The ability of ants to access different microclimates within the full vertical topsoil profile would reduce the constraints of unfavorable soil-surface conditions. Additionally, innovations in building behavior that give rise to the invasion of new habitats might subsequently facilitate adaptive radiation^42^.

The significant interaction effect of natal elevation and temperature treatment on nest size (area of soil excavated) and complexity (number of tunnels) suggests a level of local adaptation in this extended phenotypic trait. In other words, the elevation that workers originated from did not predict the size or complexity of their resulting nest, but ants excavated larger and more complex nests under conditions that were similar to their native habitat. While workers from high elevations created larger (greater area) and more complex (more tunnels) nests under the cooler temperature treatment, workers from low elevations created larger and more complex nests under the warmer temperature treatment. This result suggests that metabolic rate may be a locally adapted trait in *F. podzolica* that influences their elevational distribution. The metabolic cold adaptation hypothesis posits that cold environments (e.g. high elevations and latitudes) select for high metabolic rates, even after controlling for body size differences, and that this enables high activity levels when an organism is near its lower thermal limits^43^. Although we did not expose the ants in our experiment to temperatures near their thermal limits, a locally adapted metabolic rate may still be at play here, leading to higher excavation performance in temperatures more closely matching their original habitat.

Our results suggest that we need to consider extended phenotypes to predict how some ectothermic species respond to climate change. Organisms may be able to behaviorally modulate their exposure to extreme temperatures by creating appropriate thermal microenvironments. We hypothesize that *F. podzolica* workers produce nest architecture that helps them to buffer against thermal extremes. The apparent plasticity of nest depth raises intriguing questions about the extent to which colony life may relax selection on worker‐level metabolic traits related to climate variability. Workers’ thermal performance traits shape ant ecology and distributions at local^44–46^ and biogeographic scales^25,47,48^. However, colony‐level performance is also governed by a capacity for thermoregulation^49^ since colonies can use nest architecture to thermally manipulate larval development rates^50^ and shift colony growth rates^51^.

Behavioral plasticity may help some species mitigate the adverse impacts of climate change and thus should be an essential predictor of an organism’s climate warming vulnerability and extinction risk^52^. At a small spatial scale, elevation is the dominant factor affecting differences in mean annual surface air temperature. We expect that this altitudinal variability in climatic conditions selects for more plastic phenotypes than less climatically variable environments (such as tropical regions at lower latitudes and altitudes)^53^. In this sense, species inhabiting large altitudinal and latitudinal gradients, like *F. podzolica*, should be excellent models for future studies on the plasticity for behavioral thermoregulation under different climate change scenarios. Further, our findings suggest that nest architecture may be a plastic extended phenotypic trait in other ant species.

## Conclusions

Subterranean-nesting ants contribute to critical ecosystem services by building nests. These nests serve numerous essential functions for the colonies living within and alter soil ecosystems through aeration and the addition of nutrients. In this study, we used a laboratory approach to analyze how natal elevation and surface air temperature influence variation in nest architecture. Worker groups experiencing a warmer temperature produced deeper nests irrespective of their natal population, demonstrating that nest architecture can be a plastic response to the environment. Ants originally from high elevations excavated larger and more complex nests in a cooler temperature, while ants originally from low elevations excavated larger and more complex nests in a warmer temperature. This result suggests a level of local adaptation at play in nest architectural outcomes. Our findings suggest that a combination of plasticity and local adaptation of nest architecture contribute to the widespread intraspecific geographic success of *F. podzolica* and likely other ant species.

## Supplementary Information

Supplementary Information.

## Data Availability

Data available in the supplementary information (Sankovitz & Purcell 2021).

## References

[1] Minter NJ, Franks NR, Brown KAR (2012). Morphogenesis of an extended phenotype: Four-dimensional ant nest architecture. J. R. Soc. Interface.

[2] Dawkins R (2016). The Extended Phenotype: The Long Reach of the Gene.

[3] Tschinkel WR (2015). The architecture of subterranean ant nests: Beauty and mystery underfoot. J. Bioecon..

[4] Brian MV, Brian MV (1978). Production Ecology of Ants and Termites.

[5] De Bruyn LAL, Conacher AJ (1990). The role of termites and ants in soil modification: A review. Soil Res..

[6] Sankovitz MA, Breed MD (2019). Effects of *Formica podzolica* ant colonies on soil moisture, nitrogen, and plant communities near nests. Ecol. Entomol..

[7] Tschinkel WR (2003). Subterranean ant nests: Trace fossils past and future?. Palaeogeogr. Palaeoclimatol. Palaeoecol..

[8] Pinter-Wollman N (2015). Nest architecture shapes the collective behaviour of harvester ants. Biol. Lett..

[9] Rosengren R, Fortelius W, Lindström K, Luther A (1987). Phenology and causation of nest heating and thermoregulation in red wood ants of the *Formica rufa* group studied in coniferous forest habitats in southern Finland. Ann. Zool. Fennici.

[10] Hölldobler B, Wilson EO (1990). The Ants.

[11] Savolainen R, Vepsäläinen K (1988). A competition hierarchy among boreal ants: Impact on resource partitioning and community structure. Oikos.

[12] Frouz J, Jílková V, Sorvari J (2016). Contribution of wood ants to nutrient cycling and ecosystem function. Wood Ant Ecol. Conserv..

[13] Seeley T, Heinrich B (1981). Regulation of Temperature in the Nests of Social Insects.

[14] Hillel D (1998). Environmental Soil Physics: Fundamentals, Applications, and Environmental Considerations.

[15] Blomqvist MM, Olff H, Blaauw MB, Bongers T, Van Der Putten WH (2000). Interactions between above- and belowground biota: Importance for small-scale vegetation mosaics in a grassland ecosystem. Oikos.

[16] MacMahon JA, Mull JF, Crist TO (2000). Harvester ants (*Pogonomyrmex* spp.): Their community and ecosystem influences. Annu. Rev. Ecol. Syst..

[17] Jones CG, Lawton JH, Shachak M (1994). Organisms as ecosystem engineers. Ecosystem Management.

[18] Jouquet P, Dauber J, Lagerlöf J, Lavelle P, Lepage M (2006). Soil invertebrates as ecosystem engineers: Intended and accidental effects on soil and feedback loops. Appl. Soil Ecol..

[19] Khuong A (2016). Stigmergic construction and topochemical information shape ant nest architecture. Proc. Natl. Acad. Sci. U. S. A..

[20] Bishop TR (2019). Thermoregulatory traits combine with range shifts to alter the future of montane ant assemblages. Glob. Change Biol..

[21] Sala OE (2000). Global biodiversity scenarios for the year 2100. Science.

[22] Braschler B (2020). Realised rather than fundamental thermal niches predict site occupancy: Implications for climate change forecasting. J. Anim. Ecol..

[23] Roeder KA, Bujan J, Beurs KM, Weiser MD, Kaspari M (2021). Thermal traits predict the winners and losers under climate change: An example from North American ant communities. Ecosphere.

[24] Deutsch CA (2008). Impacts of climate warming on terrestrial ectotherms across latitude. Proc. Natl. Acad. Sci. U. S. A..

[25] Diamond SE, Sorger DM, Hulcr J, Pelini SL (2012). Who likes it hot? A global analysis of the climatic, ecological, and evolutionary determinants of warming tolerance in ants. Glob. Change Biol..

[26] Hoffmann AA, Chown SL, Clusella-Trullas S (2013). Upper thermal limits in terrestrial ectotherms: How constrained are they?. Funct. Ecol..

[27] Wilson EO (1992). The effects of complex social life on evolution and biodiversity. Oikos.

[28] Huey RB, Stevenson RD (1979). Integrating thermal physiology and ecology of ectotherms: A discussion of approaches. Am. Zool..

[29] Deslippe RJ, Savolainen R (1995). Colony foundation and polygyny in the ant *Formica podzolica*. Behav. Ecol. Sociobiol..

[30] Schneider CA, Rasband WS, Eliceiri KW (2012). NIH Image to ImageJ: 25 years of image analysis. Nat. Methods.

[31] Chambers JM, Freeny A, Heiberger RM (1992). Analysis of variance; designed experiments. Stat. Models S.

[32] Fox J (2015). Applied Regression Analysis and Generalized Linear Models.

[33] Mikheyev AS, Tschinkel WR (2004). Nest architecture of the ant *Formica pallidefulva*: Structure, costs and rules of excavation. Insectes Soc..

[34] Coppernoll-Houston D, Potter C (2018). Field measurements and satellite remote sensing of daily soil surface temperature variations in the lower Colorado desert of California. Climate.

[35] Jílková V, Cajthaml T, Frouz J (2015). Respiration in wood ant (*Formica aquilonia*) nests as affected by altitudinal and seasonal changes in temperature. Soil Biol. Biochem..

[36] Kadochová Š, Frouz J, Roces F (2017). Sun basking in red wood ants *Formica polyctena* (Hymenoptera, Formicidae): Individual behaviour and temperature-dependent respiration rates. PLoS ONE.

[37] Bollazzi M, Kronenbitter J, Roces F (2008). Soil temperature, digging behaviour, and the adaptive value of nest depth in South American species of *Acromyrmex* leaf-cutting ants. Oecologia.

[38] Stockan JA, Robinson EJH (2016). Wood Ant Ecology and Conservation.

[39] Porter SD (1988). Impact of temperature on colony growth and developmental rates of the ant, *Solenopsis invicta*. J. Insect Physiol..

[40] Lapointe SL, Serrano MS, Jones PG (1998). Microgeographic and vertical distribution of *Acromynnex landolti* (Hymenoptera: Formicidae) nests in a Neotropical Savanna. Environ. Entomol..

[41] Fowler HG (2008). Leaf-cuttings ants of the genera *Atta* and *Acromyrmex* of Paraguay (Hymenoptera: Formicidae). Mmitt. Mus. Naturkunde Berl. Dtsch. Entomol. Z..

[42] Hansell M, Hansell MH (2005). Animal Architecture.

[43] Shik JZ, Arnan X, Oms CS, Cerdá X, Boulay R (2019). Evidence for locally adaptive metabolic rates among ant populations along an elevational gradient. J. Anim. Ecol..

[44] Cerda X, Retana J, Cros S (1998). Critical thermal limits in Mediterranean ant species: Trade-off between mortality risk and foraging performance. Funct. Ecol..

[45] Kaspari M, Clay NA, Lucas J, Yanoviak SP, Kay A (2015). Thermal adaptation generates a diversity of thermal limits in a rainforest ant community. Glob. Change Biol..

[46] Talbot M (1934). Distribution of ant species in the Chicago region with reference to ecological factors and physiological toleration. Ecology.

[47] Arnan X, Blüthgen N (2015). Using ecophysiological traits to predict climatic and activity niches: Lethal temperature and water loss in Mediterranean ants: Using physiology to predict niches. Glob. Ecol. Biogeogr..

[48] Arnan X, Blüthgen N, Molowny-Horas R, Retana J (2015). Thermal characterization of European ant communities along thermal gradients and its implications for community resilience to temperature variability. Front. Ecol. Evol..

[49] Baudier KM, O’Donnell S (2016). Structure and thermal biology of subterranean army ant bivouacs in tropical montane forests. Insectes Soc..

[50] Penick CA, Tschinkel WR (2008). Thermoregulatory brood transport in the fire ant, *Solenopsis invicta*. Insectes Soc..

[51] Penick CA, Diamond SE, Sanders NJ, Dunn RR (2017). Beyond thermal limits: Comprehensive metrics of performance identify key axes of thermal adaptation in ants. Funct. Ecol..

[52] Kearney M, Shine R, Porter WP (2009). The potential for behavioral thermoregulation to buffer ‘cold-blooded’ animals against climate warming. Proc. Natl. Acad. Sci. U. S. A..

[53] Ghalambor CK, Huey RB, Martin PR, Tewksbury JJ, Wang G (2006). Are mountain passes higher in the tropics? Janzen’s hypothesis revisited. Integr. Comp. Biol..

