# Supplementary material for: Ant nest architecture is shaped by local adaptation and plastic response to temperature
Source: Sci Rep. 2021 Nov 29;11:23053. doi: 10.1038/s41598-021-02491-w (PMC8630048; doi:10.1038/s41598-021-02491-w)
Supplement: Supplementary file 1 — Supplementary Information. [file 41598_2021_2491_MOESM1_ESM.docx]

Ant nest architecture is shaped by local adaptation and plastic response to temperature

Madison Sankovitz & Jessica Purcell


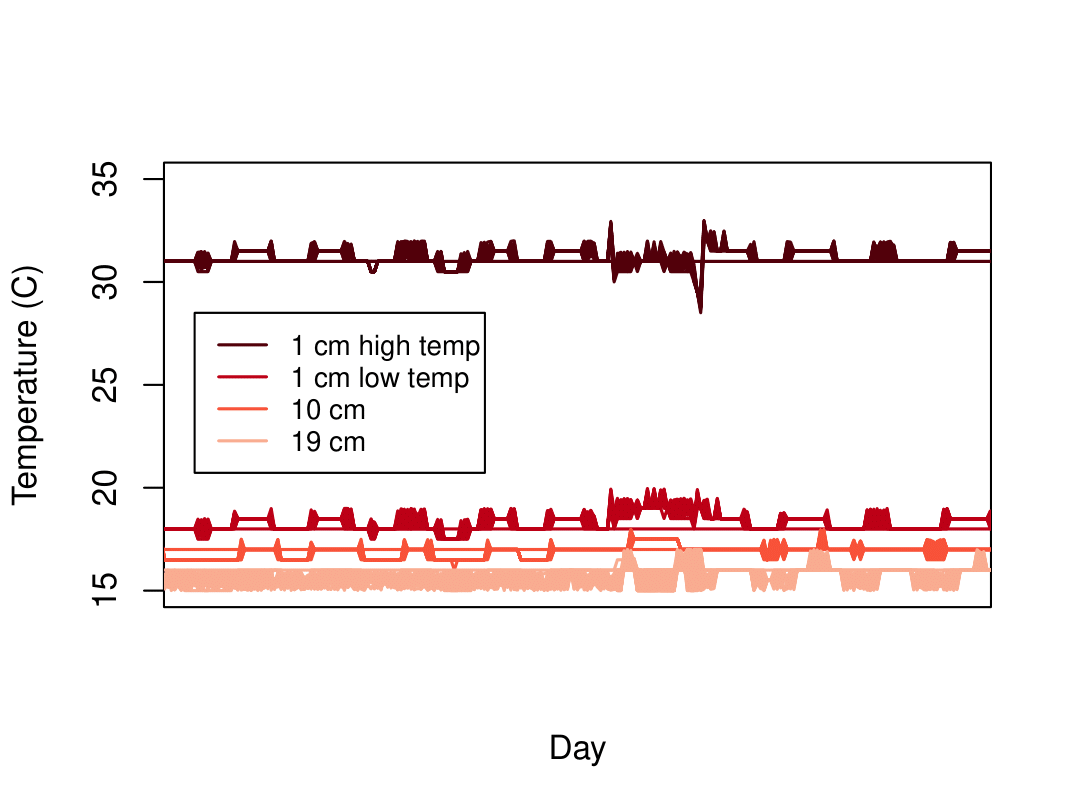


Figure S1. Cumulative iButton temperature sensor data throughout the 7-day trials. 10 and 19 cm depths experienced approximately the same temperature across treatments.

Table S1. Slope regression line per day in excavation progression plots showing nest depth, tunnel area, and the number of tunnels.

| **Nest attribute** | **Temperature** | **Elevation** | **Day 1** | **Day 2** | **Day 3** | **Day 4** | **Day 5** | **Day 6** | **Day 7** |
| --- | --- | --- | --- | --- | --- | --- | --- | --- | --- |
| Depth (cm) | Cool | Low | 3.766 | 3.247 | 1.195 | 1.008 | 0.249 | 0.308 | 0.2 |
|  |  | High | 3.373 | 2.827 | 1.825 | 1.047 | 1.298 | 0.075 | 0.410 |
|  | Warm | Low | 2.967 | 4.147 | 2.535 | 2.646 | 1.247 | 1.925 | 0.483 |
|  |  | High | 1.877 | 3.260 | 2.563 | 2.8 | 1.492 | 2.235 | 0.829 |
| Area (cm^2^) | Cool | Low | 4.875 | 2.919 | 3.591 | 2.786 | 3.627 | 2.595 | 2.762 |
|  |  | High | 3.436 | 11.044 | 6.736 | 2.862 | 3.297 | 2.553 | 6.662 |
|  | Warm | Low | 10.405 | 7.803 | 4.425 | 4.944 | 6.654 | 11.665 | 6.642 |
|  |  | High | 2.853 | 2.993 | 2.033 | 0.894 | 2.197 | 3.234 | 4.708 |
| Number of tunnels | Cool | Low | 3.167 | 1.5 | 1.583 | 1.25 | 1.167 | 0.833 | 0.5 |
|  |  | High | 3.444 | 3.112 | 2.777 | 1.111 | 0.667 | 0.557 | 0.333 |
|  | Warm | Low | 5 | 7.75 | 1.625 | 1.625 | 2.75 | 1.25 | 2.125 |
|  |  | High | 1.750 | 2.125 | 0.5 | 0.125 | 0.25 | 0.5 | 0.875 |

Table S2. Sample information and nest measurements for all colonies, including date collected, day of the trial (1-7), nest depth, area, and number of tunnels, site, elevation group, and temperature treatment. Sites: PRMP = Platt-Rogers Memorial Park; ST = Sourdough Trail; RR = Reynolds Ranch; MLOS = Mud Lake Open Space; BLRA = Brainard Lake Recreation Area.

| Date | Nest | Day | Depth (cm) | Total area (cm^2^) | Total tunnels | Elevation | Temperature | Site |
| --- | --- | --- | --- | --- | --- | --- | --- | --- |
| 7/22/19 | 1 | 1 | 10.1 | 9.294 | 2 | Low | Warm | PRMP |
| 7/22/19 | 1 | 2 | 13.1 | 11.343 | 2 | Low | Warm | PRMP |
| 7/22/19 | 1 | 3 | 13.1 | 11.696 | 3 | Low | Warm | PRMP |
| 7/22/19 | 1 | 4 | 13.1 | 12.081 | 3 | Low | Warm | PRMP |
| 7/22/19 | 1 | 5 | 13.1 | 12.728 | 3 | Low | Warm | PRMP |
| 7/22/19 | 1 | 6 | 13.1 | 12.955 | 3 | Low | Warm | PRMP |
| 7/22/19 | 1 | 7 | 13.1 | 12.89 | 3 | Low | Warm | PRMP |
| 7/22/19 | 2 | 1 | 4.7 | 2.116 | 2 | High | Warm | ST |
| 7/22/19 | 2 | 2 | 8.7 | 2.359 | 13 | High | Warm | ST |
| 7/22/19 | 2 | 3 | 17.1 | 2.367 | 13 | High | Warm | ST |
| 7/22/19 | 2 | 4 | 17.1 | 2.393 | 13 | High | Warm | ST |
| 7/22/19 | 2 | 5 | 17.1 | 2.418 | 13 | High | Warm | ST |
| 7/22/19 | 2 | 6 | 17.1 | 2.541 | 13 | High | Warm | ST |
| 7/22/19 | 2 | 7 | 17.1 | 2.575 | 13 | High | Warm | ST |
| 7/22/19 | 3 | 1 | 1.5 | 8.194 | 2 | High | Warm | ST |
| 7/22/19 | 3 | 2 | 6.5 | 10.431 | 6 | High | Warm | ST |
| 7/22/19 | 3 | 3 | 13.6 | 13.27 | 8 | High | Warm | ST |
| 7/22/19 | 3 | 4 | 13.6 | 13.729 | 8 | High | Warm | ST |
| 7/22/19 | 3 | 5 | 13.6 | 15.138 | 8 | High | Warm | ST |
| 7/22/19 | 3 | 6 | 13.6 | 15.15 | 8 | High | Warm | ST |
| 7/22/19 | 3 | 7 | 13.6 | 17.514 | 8 | High | Warm | ST |
| 7/22/19 | 4 | 1 | 4 | 4.109 | 3 | Low | Cool | PRMP |
| 7/22/19 | 4 | 2 | 8.9 | 7.974 | 3 | Low | Cool | PRMP |
| 7/22/19 | 4 | 3 | 9.1 | 18.443 | 3 | Low | Cool | PRMP |
| 7/22/19 | 4 | 4 | 9.2 | 18.896 | 3 | Low | Cool | PRMP |
| 7/22/19 | 4 | 5 | 9.2 | 19.354 | 3 | Low | Cool | PRMP |
| 7/22/19 | 4 | 6 | 9.2 | 19.226 | 3 | Low | Cool | PRMP |
| 7/22/19 | 4 | 7 | 9.2 | 21.531 | 3 | Low | Cool | PRMP |
| 7/22/19 | 5 | 1 | 7.7 | 3.932 | 4 | Low | Warm | PRMP |
| 7/22/19 | 5 | 2 | 12.9 | 12.306 | 10 | Low | Warm | PRMP |
| 7/22/19 | 5 | 3 | 12.9 | 12.455 | 10 | Low | Warm | PRMP |
| 7/22/19 | 5 | 4 | 17.1 | 13.608 | 11 | Low | Warm | PRMP |
| 7/22/19 | 5 | 5 | 17.1 | 16.126 | 11 | Low | Warm | PRMP |
| 7/22/19 | 5 | 6 | 17.1 | 21.946 | 11 | Low | Warm | PRMP |
| 7/22/19 | 5 | 7 | 17.1 | 35.789 | 11 | Low | Warm | PRMP |
| 7/22/19 | 6 | 1 | 6.9 | 0 | 14 | High | Cool | ST |
| 7/22/19 | 6 | 2 | 6.9 | 18.941 | 19 | High | Cool | ST |
| 7/22/19 | 6 | 3 | 6.9 | 26.302 | 22 | High | Cool | ST |
| 7/22/19 | 6 | 4 | 7.8 | 35.471 | 24 | High | Cool | ST |
| 7/22/19 | 6 | 5 | 10.3 | 38.15 | 25 | High | Cool | ST |
| 7/22/19 | 6 | 6 | 10.3 | 43.243 | 28 | High | Cool | ST |
| 7/22/19 | 6 | 7 | 13.7 | 48.977 | 40 | High | Cool | ST |
| 7/22/19 | 7 | 1 | 0 | 0 | 0 | Low | Warm | RR |
| 7/22/19 | 7 | 2 | 0 | 0 | 0 | Low | Warm | RR |
| 7/22/19 | 7 | 3 | 0 | 0 | 0 | Low | Warm | RR |
| 7/22/19 | 7 | 4 | 0 | 0 | 0 | Low | Warm | RR |
| 7/22/19 | 7 | 5 | 0 | 0 | 0 | Low | Warm | RR |
| 7/22/19 | 7 | 6 | 0 | 0 | 0 | Low | Warm | RR |
| 7/22/19 | 7 | 7 | 0 | 0 | 0 | Low | Warm | RR |
| 7/22/19 | 8 | 1 | 3.3 | 7.979 | 4 | Low | Cool | RR |
| 7/22/19 | 8 | 2 | 4.7 | 9.709 | 5 | Low | Cool | RR |
| 7/22/19 | 8 | 3 | 5.9 | 10.096 | 5 | Low | Cool | RR |
| 7/22/19 | 8 | 4 | 9 | 10.891 | 13 | Low | Cool | RR |
| 7/22/19 | 8 | 5 | 10 | 12.001 | 18 | Low | Cool | RR |
| 7/22/19 | 8 | 6 | 11.7 | 12.694 | 21 | Low | Cool | RR |
| 7/22/19 | 8 | 7 | 14 | 13.183 | 26 | Low | Cool | RR |
| 7/22/19 | 9 | 1 | 3.3 | 9.8 | 8 | Low | Cool | RR |
| 7/22/19 | 9 | 2 | 10.7 | 9.8 | 14 | Low | Cool | RR |
| 7/22/19 | 9 | 3 | 10.7 | 9.8 | 16 | Low | Cool | RR |
| 7/22/19 | 9 | 4 | 10.7 | 9.8 | 16 | Low | Cool | RR |
| 7/22/19 | 9 | 5 | 10.7 | 9.8 | 16 | Low | Cool | RR |
| 7/22/19 | 9 | 6 | 10.7 | 9.8 | 18 | Low | Cool | RR |
| 7/22/19 | 9 | 7 | 10.7 | 9.8 | 18 | Low | Cool | RR |
| 7/22/19 | 10 | 1 | 1.5 | 0.189 | 1 | High | Cool | ST |
| 7/22/19 | 10 | 2 | 6.1 | 17.839 | 13 | High | Cool | ST |
| 7/22/19 | 10 | 3 | 14.2 | 38.333 | 27 | High | Cool | ST |
| 7/22/19 | 10 | 4 | 16.1 | 44.109 | 28 | High | Cool | ST |
| 7/22/19 | 10 | 5 | 16.1 | 54.532 | 28 | High | Cool | ST |
| 7/22/19 | 10 | 6 | 16.1 | 57.895 | 28 | High | Cool | ST |
| 7/22/19 | 10 | 7 | 16.1 | 68.095 | 28 | High | Cool | ST |
| 7/22/19 | 11 | 1 | 4.7 | 6.263 | 6 | Low | Warm | MLOS |
| 7/22/19 | 11 | 2 | 9 | 28.877 | 13 | Low | Warm | MLOS |
| 7/22/19 | 11 | 3 | 11.3 | 27.308 | 14 | Low | Warm | MLOS |
| 7/22/19 | 11 | 4 | 11.3 | 37.048 | 21 | Low | Warm | MLOS |
| 7/22/19 | 11 | 5 | 16.6 | 46.567 | 34 | Low | Warm | MLOS |
| 7/22/19 | 11 | 6 | 18.4 | 87.746 | 36 | Low | Warm | MLOS |
| 7/22/19 | 11 | 7 | 21 | 70.702 | 37 | Low | Warm | MLOS |
| 7/22/19 | 12 | 1 | 2.6 | 4.255 | 3 | High | Warm | ST |
| 7/22/19 | 12 | 2 | 2.6 | 4.14 | 3 | High | Warm | ST |
| 7/22/19 | 12 | 3 | 3.6 | 6.606 | 5 | High | Warm | ST |
| 7/22/19 | 12 | 4 | 6 | 8.255 | 7 | High | Warm | ST |
| 7/22/19 | 12 | 5 | 8.8 | 10.264 | 14 | High | Warm | ST |
| 7/22/19 | 12 | 6 | 9 | 17.177 | 16 | High | Warm | ST |
| 7/22/19 | 12 | 7 | 10.2 | 23.186 | 18 | High | Warm | ST |
| 7/22/19 | 13 | 1 | 0 | 0 | 0 | High | Warm | ST |
| 7/22/19 | 13 | 2 | 4.5 | 3.443 | 1 | High | Warm | ST |
| 7/22/19 | 13 | 3 | 4.5 | 4.417 | 1 | High | Warm | ST |
| 7/22/19 | 13 | 4 | 4.5 | 6.605 | 1 | High | Warm | ST |
| 7/22/19 | 13 | 5 | 4.5 | 7.498 | 1 | High | Warm | ST |
| 7/22/19 | 13 | 6 | 17.6 | 20.382 | 4 | High | Warm | ST |
| 7/22/19 | 13 | 7 | 17.6 | 23.563 | 7 | High | Warm | ST |
| 7/22/19 | 14 | 1 | 2.5 | 1.641 | 3 | High | Warm | BLRA |
| 7/22/19 | 14 | 2 | 16.2 | 4.506 | 4 | High | Warm | BLRA |
| 7/22/19 | 14 | 3 | 16.2 | 4.981 | 4 | High | Warm | BLRA |
| 7/22/19 | 14 | 4 | 16.2 | 5.947 | 4 | High | Warm | BLRA |
| 7/22/19 | 14 | 5 | 16.2 | 6.038 | 4 | High | Warm | BLRA |
| 7/22/19 | 14 | 6 | 16.2 | 6.45 | 4 | High | Warm | BLRA |
| 7/22/19 | 14 | 7 | 16.2 | 8.679 | 4 | High | Warm | BLRA |
| 7/22/19 | 15 | 1 | 0 | 0 | 0 | Low | Warm | MLOS |
| 7/22/19 | 15 | 2 | 0 | 0 | 0 | Low | Warm | MLOS |
| 7/22/19 | 15 | 3 | 0 | 0 | 0 | Low | Warm | MLOS |
| 7/22/19 | 15 | 4 | 0 | 0 | 0 | Low | Warm | MLOS |
| 7/22/19 | 15 | 5 | 0 | 0 | 0 | Low | Warm | MLOS |
| 7/22/19 | 15 | 6 | 0 | 0 | 0 | Low | Warm | MLOS |
| 7/22/19 | 15 | 7 | 0 | 0 | 0 | Low | Warm | MLOS |
| 7/22/19 | 16 | 1 | 1.7 | 0.303 | 1 | Low | Cool | MLOS |
| 7/22/19 | 16 | 2 | 5.3 | 6.634 | 2 | Low | Cool | MLOS |
| 7/22/19 | 16 | 3 | 7.2 | 7.48 | 3 | Low | Cool | MLOS |
| 7/22/19 | 16 | 4 | 7.2 | 9.702 | 5 | Low | Cool | MLOS |
| 7/22/19 | 16 | 5 | 7.2 | 13.409 | 6 | Low | Cool | MLOS |
| 7/22/19 | 16 | 6 | 8.6 | 18.308 | 7 | Low | Cool | MLOS |
| 7/22/19 | 16 | 7 | 9.2 | 21.014 | 7 | Low | Cool | MLOS |
| 7/22/19 | 17 | 1 | 2.2 | 6.073 | 2 | Low | Cool | MLOS |
| 7/22/19 | 17 | 2 | 5.1 | 8.757 | 2 | Low | Cool | MLOS |
| 7/22/19 | 17 | 3 | 7.2 | 10.956 | 5 | Low | Cool | MLOS |
| 7/22/19 | 17 | 4 | 7.2 | 11.699 | 6 | Low | Cool | MLOS |
| 7/22/19 | 17 | 5 | 7.2 | 12.42 | 7 | Low | Cool | MLOS |
| 7/22/19 | 17 | 6 | 8.5 | 13.485 | 10 | Low | Cool | MLOS |
| 7/22/19 | 17 | 7 | 8.5 | 14.967 | 10 | Low | Cool | MLOS |
| 7/22/19 | 18 | 1 | 0 | 17.9 | 1 | High | Warm | BLRA |
| 7/22/19 | 18 | 2 | 4.3 | 17.9 | 1 | High | Warm | BLRA |
| 7/22/19 | 18 | 3 | 6.8 | 17.9 | 1 | High | Warm | BLRA |
| 7/22/19 | 18 | 4 | 11.9 | 17.9 | 1 | High | Warm | BLRA |
| 7/22/19 | 18 | 5 | 11.9 | 17.9 | 1 | High | Warm | BLRA |
| 7/22/19 | 18 | 6 | 11.9 | 17.9 | 1 | High | Warm | BLRA |
| 7/22/19 | 18 | 7 | 11.9 | 17.9 | 1 | High | Warm | BLRA |
| 7/22/19 | 19 | 1 | 1.5 | 0 | 2 | High | Cool | BLRA |
| 7/22/19 | 19 | 2 | 1.5 | 0 | 2 | High | Cool | BLRA |
| 7/22/19 | 19 | 3 | 1.5 | 0 | 2 | High | Cool | BLRA |
| 7/22/19 | 19 | 4 | 1.5 | 0 | 2 | High | Cool | BLRA |
| 7/22/19 | 19 | 5 | 4.3 | 0.82 | 3 | High | Cool | BLRA |
| 7/22/19 | 19 | 6 | 4.3 | 2.319 | 4 | High | Cool | BLRA |
| 7/22/19 | 19 | 7 | 5.2 | 5.178 | 6 | High | Cool | BLRA |
| 7/22/19 | 20 | 1 | 0 | 0 | 0 | High | Cool | BLRA |
| 7/22/19 | 20 | 2 | 0 | 0 | 0 | High | Cool | BLRA |
| 7/22/19 | 20 | 3 | 0 | 0 | 0 | High | Cool | BLRA |
| 7/22/19 | 20 | 4 | 0 | 0 | 0 | High | Cool | BLRA |
| 7/22/19 | 20 | 5 | 0 | 0 | 0 | High | Cool | BLRA |
| 7/22/19 | 20 | 6 | 0 | 0 | 0 | High | Cool | BLRA |
| 7/22/19 | 20 | 7 | 0 | 0 | 0 | High | Cool | BLRA |
| 7/30/19 | 21 | 1 | 3.6 | 0.046 | 2 | High | Warm | ST |
| 7/30/19 | 21 | 2 | 4.1 | 0.095 | 2 | High | Warm | ST |
| 7/30/19 | 21 | 3 | 3.9 | 0.091 | 2 | High | Warm | ST |
| 7/30/19 | 21 | 4 | 5.6 | 1.546 | 3 | High | Warm | ST |
| 7/30/19 | 21 | 5 | 9.2 | 5.952 | 5 | High | Warm | ST |
| 7/30/19 | 21 | 6 | 12.9 | 9.378 | 6 | High | Warm | ST |
| 7/30/19 | 21 | 7 | 17 | 12.515 | 6 | High | Warm | ST |
| 7/30/19 | 22 | 1 | 7.6 | 3.876 | 2 | High | Warm | BLRA |
| 7/30/19 | 22 | 2 | 7.3 | 4.101 | 2 | High | Warm | BLRA |
| 7/30/19 | 22 | 3 | 13.3 | 11.166 | 4 | High | Warm | BLRA |
| 7/30/19 | 22 | 4 | 13.1 | 11.346 | 4 | High | Warm | BLRA |
| 7/30/19 | 22 | 5 | 13.3 | 11.419 | 4 | High | Warm | BLRA |
| 7/30/19 | 22 | 6 | 13.1 | 12.254 | 4 | High | Warm | BLRA |
| 7/30/19 | 22 | 7 | 13.8 | 13.754 | 4 | High | Warm | BLRA |
| 7/30/19 | 23 | 1 | 0 | 10 | 1 | High | Warm | BLRA |
| 7/30/19 | 23 | 2 | 1.4 | 10 | 1 | High | Warm | BLRA |
| 7/30/19 | 23 | 3 | 6 | 10 | 1 | High | Warm | BLRA |
| 7/30/19 | 23 | 4 | 15.1 | 10 | 1 | High | Warm | BLRA |
| 7/30/19 | 23 | 5 | 16.3 | 10 | 1 | High | Warm | BLRA |
| 7/30/19 | 23 | 6 | 16.3 | 10 | 1 | High | Warm | BLRA |
| 7/30/19 | 23 | 7 | 16.3 | 10 | 1 | High | Warm | BLRA |
| 7/30/19 | 24 | 1 | 0 | 2.869 | 1 | High | Warm | BLRA |
| 7/30/19 | 24 | 2 | 8.9 | 14.709 | 1 | High | Warm | BLRA |
| 7/30/19 | 24 | 3 | 9 | 17.571 | 1 | High | Warm | BLRA |
| 7/30/19 | 24 | 4 | 8.9 | 18.451 | 1 | High | Warm | BLRA |
| 7/30/19 | 24 | 5 | 9.1 | 23.782 | 1 | High | Warm | BLRA |
| 7/30/19 | 24 | 6 | 9 | 25.206 | 1 | High | Warm | BLRA |
| 7/30/19 | 24 | 7 | 8.9 | 35.368 | 1 | High | Warm | BLRA |
| 7/30/19 | 25 | 1 | 0 | 0 | 0 | High | Warm | ST |
| 7/30/19 | 25 | 2 | 0 | 0 | 0 | High | Warm | ST |
| 7/30/19 | 25 | 3 | 0 | 0 | 0 | High | Warm | ST |
| 7/30/19 | 25 | 4 | 0 | 0 | 0 | High | Warm | ST |
| 7/30/19 | 25 | 5 | 0 | 0 | 0 | High | Warm | ST |
| 7/30/19 | 25 | 6 | 0 | 0 | 0 | High | Warm | ST |
| 7/30/19 | 25 | 7 | 0 | 0 | 0 | High | Warm | ST |
| 7/30/19 | 26 | 1 | 5 | 1.786 | 4 | Low | Cool | PRMP |
| 7/30/19 | 26 | 2 | 5.1 | 2.061 | 4 | Low | Cool | PRMP |
| 7/30/19 | 26 | 3 | 6.8 | 9.558 | 8 | Low | Cool | PRMP |
| 7/30/19 | 26 | 4 | 9.1 | 13.533 | 10 | Low | Cool | PRMP |
| 7/30/19 | 26 | 5 | 9.7 | 15.538 | 10 | Low | Cool | PRMP |
| 7/30/19 | 26 | 6 | 9.7 | 17.93 | 11 | Low | Cool | PRMP |
| 7/30/19 | 26 | 7 | 9.7 | 24.882 | 12 | Low | Cool | PRMP |
| 7/30/19 | 27 | 1 | 3.8 | 1.518 | 4 | Low | Cool | PRMP |
| 7/30/19 | 27 | 2 | 4.5 | 2.221 | 5 | Low | Cool | PRMP |
| 7/30/19 | 27 | 3 | 6.5 | 2.822 | 5 | Low | Cool | PRMP |
| 7/30/19 | 27 | 4 | 6.2 | 3.164 | 6 | Low | Cool | PRMP |
| 7/30/19 | 27 | 5 | 7.1 | 3.21 | 7 | Low | Cool | PRMP |
| 7/30/19 | 27 | 6 | 7.1 | 4.472 | 7 | Low | Cool | PRMP |
| 7/30/19 | 27 | 7 | 7.2 | 4.618 | 7 | Low | Cool | PRMP |
| 7/30/19 | 28 | 1 | 14.6 | 5.368 | 3 | Low | Cool | MLOS |
| 7/30/19 | 28 | 2 | 19 | 7.077 | 5 | Low | Cool | MLOS |
| 7/30/19 | 28 | 3 | 19 | 10.721 | 5 | Low | Cool | MLOS |
| 7/30/19 | 28 | 4 | 19 | 17.616 | 5 | Low | Cool | MLOS |
| 7/30/19 | 28 | 5 | 19 | 33.774 | 5 | Low | Cool | MLOS |
| 7/30/19 | 28 | 6 | 19 | 34.074 | 5 | Low | Cool | MLOS |
| 7/30/19 | 28 | 7 | 19 | 42.195 | 5 | Low | Cool | MLOS |
| 7/30/19 | 29 | 1 | 4.8 | 5.195 | 4 | Low | Cool | RR |
| 7/30/19 | 29 | 2 | 4.8 | 5.212 | 4 | Low | Cool | RR |
| 7/30/19 | 29 | 3 | 4.8 | 5.265 | 4 | Low | Cool | RR |
| 7/30/19 | 29 | 4 | 4.8 | 5.336 | 4 | Low | Cool | RR |
| 7/30/19 | 29 | 5 | 4.8 | 5.463 | 4 | Low | Cool | RR |
| 7/30/19 | 29 | 6 | 4.8 | 5.49 | 4 | Low | Cool | RR |
| 7/30/19 | 29 | 7 | 4.8 | 5.922 | 4 | Low | Cool | RR |
| 7/30/19 | 30 | 1 | 0 | 15.8 | 1 | Low | Cool | RR |
| 7/30/19 | 30 | 2 | 1.5 | 15.8 | 1 | Low | Cool | RR |
| 7/30/19 | 30 | 3 | 2.9 | 15.8 | 1 | Low | Cool | RR |
| 7/30/19 | 30 | 4 | 7 | 15.8 | 1 | Low | Cool | RR |
| 7/30/19 | 30 | 5 | 7 | 15.8 | 1 | Low | Cool | RR |
| 7/30/19 | 30 | 6 | 7 | 15.8 | 1 | Low | Cool | RR |
| 7/30/19 | 30 | 7 | 7 | 15.8 | 1 | Low | Cool | RR |
| 7/30/19 | 31 | 1 | 0 | 34.9 | 7 | Low | Warm | RR |
| 7/30/19 | 31 | 2 | 1.8 | 34.9 | 7 | Low | Warm | RR |
| 7/30/19 | 31 | 3 | 8 | 34.9 | 7 | Low | Warm | RR |
| 7/30/19 | 31 | 4 | 12.9 | 34.9 | 7 | Low | Warm | RR |
| 7/30/19 | 31 | 5 | 14.7 | 34.9 | 7 | Low | Warm | RR |
| 7/30/19 | 31 | 6 | 14.7 | 34.9 | 7 | Low | Warm | RR |
| 7/30/19 | 31 | 7 | 14.7 | 34.9 | 7 | Low | Warm | RR |
| 7/30/19 | 32 | 1 | 4.7 | 35.9 | 2 | Low | Warm | MLOS |
| 7/30/19 | 32 | 2 | 5.9 | 35.9 | 3 | Low | Warm | MLOS |
| 7/30/19 | 32 | 3 | 8 | 35.9 | 4 | Low | Warm | MLOS |
| 7/30/19 | 32 | 4 | 9.7 | 35.9 | 4 | Low | Warm | MLOS |
| 7/30/19 | 32 | 5 | 11.7 | 35.9 | 4 | Low | Warm | MLOS |
| 7/30/19 | 32 | 6 | 13.4 | 35.9 | 4 | Low | Warm | MLOS |
| 7/30/19 | 32 | 7 | 15.8 | 35.9 | 6 | Low | Warm | MLOS |
| 7/30/19 | 33 | 1 | 0 | 33.1 | 6 | Low | Warm | PRMP |
| 7/30/19 | 33 | 2 | 8.3 | 33.1 | 6 | Low | Warm | PRMP |
| 7/30/19 | 33 | 3 | 13 | 33.1 | 6 | Low | Warm | PRMP |
| 7/30/19 | 33 | 4 | 13 | 33.1 | 6 | Low | Warm | PRMP |
| 7/30/19 | 33 | 5 | 13 | 33.1 | 6 | Low | Warm | PRMP |
| 7/30/19 | 33 | 6 | 13 | 33.1 | 6 | Low | Warm | PRMP |
| 7/30/19 | 33 | 7 | 13 | 33.1 | 6 | Low | Warm | PRMP |
| 7/30/19 | 34 | 1 | 0 | 11.7 | 8 | Low | Warm | MLOS |
| 7/30/19 | 34 | 2 | 1.3 | 11.7 | 8 | Low | Warm | MLOS |
| 7/30/19 | 34 | 3 | 3.6 | 11.7 | 8 | Low | Warm | MLOS |
| 7/30/19 | 34 | 4 | 6.8 | 11.7 | 8 | Low | Warm | MLOS |
| 7/30/19 | 34 | 5 | 7 | 11.7 | 8 | Low | Warm | MLOS |
| 7/30/19 | 34 | 6 | 15.3 | 11.7 | 8 | Low | Warm | MLOS |
| 7/30/19 | 34 | 7 | 15.3 | 11.7 | 8 | Low | Warm | MLOS |
| 7/30/19 | 35 | 1 | 0 | 31.3 | 7 | Low | Warm | RR |
| 7/30/19 | 35 | 2 | 6.9 | 31.3 | 7 | Low | Warm | RR |
| 7/30/19 | 35 | 3 | 7.5 | 31.3 | 7 | Low | Warm | RR |
| 7/30/19 | 35 | 4 | 16 | 31.3 | 7 | Low | Warm | RR |
| 7/30/19 | 35 | 5 | 16 | 31.3 | 7 | Low | Warm | RR |
| 7/30/19 | 35 | 6 | 16 | 31.3 | 7 | Low | Warm | RR |
| 7/30/19 | 35 | 7 | 16 | 31.3 | 7 | Low | Warm | RR |
| 7/30/19 | 36 | 1 | 0 | 0 | 0 | High | Cool | ST |
| 7/30/19 | 36 | 2 | 0 | 0 | 0 | High | Cool | ST |
| 7/30/19 | 36 | 3 | 0 | 0 | 0 | High | Cool | ST |
| 7/30/19 | 36 | 4 | 0 | 0 | 0 | High | Cool | ST |
| 7/30/19 | 36 | 5 | 0 | 0 | 0 | High | Cool | ST |
| 7/30/19 | 36 | 6 | 0 | 0 | 0 | High | Cool | ST |
| 7/30/19 | 36 | 7 | 0 | 0 | 0 | High | Cool | ST |
| 7/30/19 | 37 | 1 | 3.6 | 0.655 | 3 | High | Cool | ST |
| 7/30/19 | 37 | 2 | 4.3 | 1.313 | 4 | High | Cool | ST |
| 7/30/19 | 37 | 3 | 7 | 5.682 | 9 | High | Cool | ST |
| 7/30/19 | 37 | 4 | 7 | 7.014 | 9 | High | Cool | ST |
| 7/30/19 | 37 | 5 | 7 | 8.031 | 9 | High | Cool | ST |
| 7/30/19 | 37 | 6 | 7 | 10.569 | 9 | High | Cool | ST |
| 7/30/19 | 37 | 7 | 7 | 12.889 | 9 | High | Cool | ST |
| 7/30/19 | 38 | 1 | 3.6 | 0.105 | 5 | High | Cool | BLRA |
| 7/30/19 | 38 | 2 | 5.2 | 3.96 | 6 | High | Cool | BLRA |
| 7/30/19 | 38 | 3 | 7 | 5.236 | 6 | High | Cool | BLRA |
| 7/30/19 | 38 | 4 | 8.8 | 6.418 | 6 | High | Cool | BLRA |
| 7/30/19 | 38 | 5 | 12.6 | 9.036 | 7 | High | Cool | BLRA |
| 7/30/19 | 38 | 6 | 12.4 | 9.423 | 7 | High | Cool | BLRA |
| 7/30/19 | 38 | 7 | 12.7 | 11.251 | 7 | High | Cool | BLRA |
| 7/30/19 | 39 | 1 | 3.9 | 16.923 | 3 | High | Cool | BLRA |
| 7/30/19 | 39 | 2 | 13.5 | 20.282 | 10 | High | Cool | BLRA |
| 7/30/19 | 39 | 3 | 15.2 | 26.465 | 15 | High | Cool | BLRA |
| 7/30/19 | 39 | 4 | 19.1 | 28.44 | 22 | High | Cool | BLRA |
| 7/30/19 | 39 | 5 | 22.1 | 43.321 | 25 | High | Cool | BLRA |
| 7/30/19 | 39 | 6 | 23.3 | 47.48 | 29 | High | Cool | BLRA |
| 7/30/19 | 39 | 7 | 23.3 | 74.603 | 30 | High | Cool | BLRA |
| 7/30/19 | 40 | 1 | 4.6 | 0 | 5 | High | Cool | BLRA |
| 7/30/19 | 40 | 2 | 6.5 | 7.756 | 7 | High | Cool | BLRA |
| 7/30/19 | 40 | 3 | 10.2 | 14.065 | 7 | High | Cool | BLRA |
| 7/30/19 | 40 | 4 | 12.1 | 14.333 | 8 | High | Cool | BLRA |
| 7/30/19 | 40 | 5 | 14.4 | 15.105 | 8 | High | Cool | BLRA |
| 7/30/19 | 40 | 6 | 14.2 | 16.838 | 8 | High | Cool | BLRA |
| 7/30/19 | 40 | 7 | 14 | 40.042 | 8 | High | Cool | BLRA |
| 8/9/19 | 41 | 1 | 3.7 | 1.232 | 2 | High | Warm | ST |
| 8/9/19 | 41 | 2 | 3.7 | 1.277 | 2 | High | Warm | ST |
| 8/9/19 | 41 | 3 | 3.7 | 1.292 | 2 | High | Warm | ST |
| 8/9/19 | 41 | 4 | 3.8 | 1.397 | 2 | High | Warm | ST |
| 8/9/19 | 41 | 5 | 7.6 | 4.548 | 2 | High | Warm | ST |
| 8/9/19 | 41 | 6 | 10.8 | 8.069 | 2 | High | Warm | ST |
| 8/9/19 | 41 | 7 | 16.7 | 18.413 | 6 | High | Warm | ST |
| 8/9/19 | 42 | 1 | 8.4 | 21.761 | 8 | Low | Warm | PRMP |
| 8/9/19 | 42 | 2 | 9.8 | 25.059 | 10 | Low | Warm | PRMP |
| 8/9/19 | 42 | 3 | 11.7 | 30.717 | 12 | Low | Warm | PRMP |
| 8/9/19 | 42 | 4 | 13.6 | 34.616 | 12 | Low | Warm | PRMP |
| 8/9/19 | 42 | 5 | 15.8 | 37.341 | 12 | Low | Warm | PRMP |
| 8/9/19 | 42 | 6 | 18 | 42.57 | 13 | Low | Warm | PRMP |
| 8/9/19 | 42 | 7 | 18.8 | 47.804 | 13 | Low | Warm | PRMP |
| 8/9/19 | 43 | 1 | 0 | 0 | 0 | Low | Warm | PRMP |
| 8/9/19 | 43 | 2 | 0 | 0 | 0 | Low | Warm | PRMP |
| 8/9/19 | 43 | 3 | 0 | 0 | 0 | Low | Warm | PRMP |
| 8/9/19 | 43 | 4 | 0 | 0 | 0 | Low | Warm | PRMP |
| 8/9/19 | 43 | 5 | 0 | 0 | 0 | Low | Warm | PRMP |
| 8/9/19 | 43 | 6 | 0 | 0 | 0 | Low | Warm | PRMP |
| 8/9/19 | 43 | 7 | 0 | 0 | 0 | Low | Warm | PRMP |
| 8/9/19 | 44 | 1 | 0 | 0 | 0 | High | Warm | BLRA |
| 8/9/19 | 44 | 2 | 0 | 0 | 0 | High | Warm | BLRA |
| 8/9/19 | 44 | 3 | 0 | 0 | 0 | High | Warm | BLRA |
| 8/9/19 | 44 | 4 | 0 | 0 | 0 | High | Warm | BLRA |
| 8/9/19 | 44 | 5 | 0 | 0 | 0 | High | Warm | BLRA |
| 8/9/19 | 44 | 6 | 0 | 0 | 0 | High | Warm | BLRA |
| 8/9/19 | 44 | 7 | 0 | 0 | 0 | High | Warm | BLRA |
| 8/9/19 | 45 | 1 | 0 | 18.3 | 1 | High | Warm | BLRA |
| 8/9/19 | 45 | 2 | 1.1 | 18.3 | 1 | High | Warm | BLRA |
| 8/9/19 | 45 | 3 | 5.1 | 18.3 | 1 | High | Warm | BLRA |
| 8/9/19 | 45 | 4 | 14.2 | 18.3 | 1 | High | Warm | BLRA |
| 8/9/19 | 45 | 5 | 16.5 | 18.3 | 1 | High | Warm | BLRA |
| 8/9/19 | 45 | 6 | 16.5 | 18.3 | 1 | High | Warm | BLRA |
| 8/9/19 | 45 | 7 | 16.5 | 18.3 | 1 | High | Warm | BLRA |
| 8/9/19 | 46 | 1 | 3.1 | 0.95 | 3 | High | Cool | ST |
| 8/9/19 | 46 | 2 | 4.9 | 6.373 | 6 | High | Cool | ST |
| 8/9/19 | 46 | 3 | 5.2 | 10.798 | 7 | High | Cool | ST |
| 8/9/19 | 46 | 4 | 6.2 | 11.087 | 8 | High | Cool | ST |
| 8/9/19 | 46 | 5 | 6.2 | 14.383 | 9 | High | Cool | ST |
| 8/9/19 | 46 | 6 | 6.2 | 15.838 | 9 | High | Cool | ST |
| 8/9/19 | 46 | 7 | 6.2 | 15.593 | 9 | High | Cool | ST |
| 8/9/19 | 47 | 1 | 3.6 | 3.382 | 1 | High | Cool | ST |
| 8/9/19 | 47 | 2 | 7.8 | 10.557 | 2 | High | Cool | ST |
| 8/9/19 | 47 | 3 | 7.8 | 11.016 | 2 | High | Cool | ST |
| 8/9/19 | 47 | 4 | 7.8 | 11.187 | 2 | High | Cool | ST |
| 8/9/19 | 47 | 5 | 7.8 | 11.995 | 2 | High | Cool | ST |
| 8/9/19 | 47 | 6 | 7.8 | 12.16 | 2 | High | Cool | ST |
| 8/9/19 | 47 | 7 | 7.8 | 12.294 | 2 | High | Cool | ST |
| 8/9/19 | 48 | 1 | 0 | 14.8 | 1 | Low | Cool | PRMP |
| 8/9/19 | 48 | 2 | 7.1 | 14.8 | 1 | Low | Cool | PRMP |
| 8/9/19 | 48 | 3 | 8.6 | 14.8 | 1 | Low | Cool | PRMP |
| 8/9/19 | 48 | 4 | 8.6 | 14.8 | 1 | Low | Cool | PRMP |
| 8/9/19 | 48 | 5 | 8.6 | 14.8 | 1 | Low | Cool | PRMP |
| 8/9/19 | 48 | 6 | 8.6 | 14.8 | 1 | Low | Cool | PRMP |
| 8/9/19 | 48 | 7 | 8.6 | 14.8 | 1 | Low | Cool | PRMP |
| 8/9/19 | 49 | 1 | 0 | 0 | 0 | Low | Cool | PRMP |
| 8/9/19 | 49 | 2 | 6.6 | 4.579 | 3 | Low | Cool | PRMP |
| 8/9/19 | 49 | 3 | 8.1 | 11.95 | 4 | Low | Cool | PRMP |
| 8/9/19 | 49 | 4 | 12.9 | 24.883 | 5 | Low | Cool | PRMP |
| 8/9/19 | 49 | 5 | 14 | 44.641 | 11 | Low | Cool | PRMP |
| 8/9/19 | 49 | 6 | 13.6 | 49.349 | 11 | Low | Cool | PRMP |
| 8/9/19 | 49 | 7 | 13.6 | 50.922 | 11 | Low | Cool | PRMP |
| 8/9/19 | 50 | 1 | 5.2 | 1.123 | 2 | Low | Cool | RR |
| 8/9/19 | 50 | 2 | 9.2 | 8.76 | 4 | Low | Cool | RR |
| 8/9/19 | 50 | 3 | 11.1 | 14.841 | 4 | Low | Cool | RR |
| 8/9/19 | 50 | 4 | 11.2 | 15.13 | 4 | Low | Cool | RR |
| 8/9/19 | 50 | 5 | 11.1 | 13.003 | 4 | Low | Cool | RR |
| 8/9/19 | 50 | 6 | 11.2 | 14.962 | 4 | Low | Cool | RR |
| 8/9/19 | 50 | 7 | 11.1 | 15.663 | 4 | Low | Cool | RR |
| 8/9/19 | 51 | 1 | 0 | 0 | 6 | Low | Warm | RR |
| 8/9/19 | 51 | 2 | 10.7 | 4.213 | 6 | Low | Warm | RR |
| 8/9/19 | 51 | 3 | 15.3 | 6.367 | 6 | Low | Warm | RR |
| 8/9/19 | 51 | 4 | 16.9 | 9.738 | 6 | Low | Warm | RR |
| 8/9/19 | 51 | 5 | 16.9 | 12.26 | 6 | Low | Warm | RR |
| 8/9/19 | 51 | 6 | 16.9 | 16.601 | 6 | Low | Warm | RR |
| 8/9/19 | 51 | 7 | 16.9 | 18.803 | 6 | Low | Warm | RR |
| 8/9/19 | 52 | 1 | 0 | 0 | 1 | Low | Warm | RR |
| 8/9/19 | 52 | 2 | 4.6 | 3.027 | 2 | Low | Warm | RR |
| 8/9/19 | 52 | 3 | 9.8 | 14.181 | 5 | Low | Warm | RR |
| 8/9/19 | 52 | 4 | 15 | 21.675 | 6 | Low | Warm | RR |
| 8/9/19 | 52 | 5 | 15.8 | 26.779 | 7 | Low | Warm | RR |
| 8/9/19 | 52 | 6 | 15.8 | 28.902 | 9 | Low | Warm | RR |
| 8/9/19 | 52 | 7 | 15.8 | 31.454 | 9 | Low | Warm | RR |
| 8/9/19 | 53 | 1 | 0 | 16.9 | 1 | High | Warm | ST |
| 8/9/19 | 53 | 2 | 1 | 16.9 | 1 | High | Warm | ST |
| 8/9/19 | 53 | 3 | 3.1 | 16.9 | 1 | High | Warm | ST |
| 8/9/19 | 53 | 4 | 13.7 | 16.9 | 1 | High | Warm | ST |
| 8/9/19 | 53 | 5 | 17.6 | 16.9 | 1 | High | Warm | ST |
| 8/9/19 | 53 | 6 | 17.6 | 16.9 | 1 | High | Warm | ST |
| 8/9/19 | 53 | 7 | 17.6 | 16.9 | 1 | High | Warm | ST |
| 8/9/19 | 54 | 1 | 0 | 11.2 | 1 | High | Warm | BLRA |
| 8/9/19 | 54 | 2 | 1.5 | 11.2 | 1 | High | Warm | BLRA |
| 8/9/19 | 54 | 3 | 2.1 | 11.2 | 1 | High | Warm | BLRA |
| 8/9/19 | 54 | 4 | 3.5 | 11.2 | 1 | High | Warm | BLRA |
| 8/9/19 | 54 | 5 | 6.3 | 11.2 | 1 | High | Warm | BLRA |
| 8/9/19 | 54 | 6 | 17.6 | 11.2 | 1 | High | Warm | BLRA |
| 8/9/19 | 54 | 7 | 17.6 | 11.2 | 1 | High | Warm | BLRA |
| 8/9/19 | 55 | 1 | 0 | 23.169 | 8 | Low | Warm | MLOS |
| 8/9/19 | 55 | 2 | 1.3 | 28.265 | 8 | Low | Warm | MLOS |
| 8/9/19 | 55 | 3 | 1.6 | 30.217 | 8 | Low | Warm | MLOS |
| 8/9/19 | 55 | 4 | 2.2 | 33.909 | 8 | Low | Warm | MLOS |
| 8/9/19 | 55 | 5 | 4.7 | 35.202 | 8 | Low | Warm | MLOS |
| 8/9/19 | 55 | 6 | 13.8 | 48.782 | 8 | Low | Warm | MLOS |
| 8/9/19 | 55 | 7 | 13.8 | 56.771 | 8 | Low | Warm | MLOS |
| 8/9/19 | 56 | 1 | 4.9 | 18.768 | 8 | High | Cool | BLRA |
| 8/9/19 | 56 | 2 | 8.8 | 34.618 | 9 | High | Cool | BLRA |
| 8/9/19 | 56 | 3 | 8.8 | 37.077 | 9 | High | Cool | BLRA |
| 8/9/19 | 56 | 4 | 8.8 | 38.926 | 9 | High | Cool | BLRA |
| 8/9/19 | 56 | 5 | 8.8 | 39.575 | 9 | High | Cool | BLRA |
| 8/9/19 | 56 | 6 | 8.8 | 41.212 | 9 | High | Cool | BLRA |
| 8/9/19 | 56 | 7 | 8.8 | 41.808 | 9 | High | Cool | BLRA |
| 8/9/19 | 57 | 1 | 0 | 27.1 | 2 | High | Cool | BLRA |
| 8/9/19 | 57 | 2 | 2.8 | 27.1 | 2 | High | Cool | BLRA |
| 8/9/19 | 57 | 3 | 4.7 | 27.1 | 2 | High | Cool | BLRA |
| 8/9/19 | 57 | 4 | 4.7 | 27.1 | 2 | High | Cool | BLRA |
| 8/9/19 | 57 | 5 | 4.7 | 27.1 | 2 | High | Cool | BLRA |
| 8/9/19 | 57 | 6 | 4.7 | 27.1 | 2 | High | Cool | BLRA |
| 8/9/19 | 57 | 7 | 4.7 | 27.1 | 2 | High | Cool | BLRA |
| 8/9/19 | 58 | 1 | 0 | 0 | 0 | High | Cool | ST |
| 8/9/19 | 58 | 2 | 0 | 0 | 0 | High | Cool | ST |
| 8/9/19 | 58 | 3 | 0 | 0 | 0 | High | Cool | ST |
| 8/9/19 | 58 | 4 | 0 | 0 | 0 | High | Cool | ST |
| 8/9/19 | 58 | 5 | 0 | 0 | 0 | High | Cool | ST |
| 8/9/19 | 58 | 6 | 0 | 0 | 0 | High | Cool | ST |
| 8/9/19 | 58 | 7 | 0 | 0 | 0 | High | Cool | ST |
| 8/9/19 | 59 | 1 | 0 | 0 | 0 | Low | Cool | MLOS |
| 8/9/19 | 59 | 2 | 0 | 0 | 0 | Low | Cool | MLOS |
| 8/9/19 | 59 | 3 | 0 | 0 | 0 | Low | Cool | MLOS |
| 8/9/19 | 59 | 4 | 0 | 0 | 0 | Low | Cool | MLOS |
| 8/9/19 | 59 | 5 | 0 | 0 | 0 | Low | Cool | MLOS |
| 8/9/19 | 59 | 6 | 0 | 0 | 0 | Low | Cool | MLOS |
| 8/9/19 | 59 | 7 | 0 | 0 | 0 | Low | Cool | MLOS |
| 8/9/19 | 60 | 1 | 4.8 | 1.881 | 3 | Low | Cool | MLOS |
| 8/9/19 | 60 | 2 | 5.6 | 2.281 | 5 | Low | Cool | MLOS |
| 8/9/19 | 60 | 3 | 7 | 4.269 | 13 | Low | Cool | MLOS |
| 8/9/19 | 60 | 4 | 7 | 5.293 | 13 | Low | Cool | MLOS |
| 8/9/19 | 60 | 5 | 7 | 5.562 | 13 | Low | Cool | MLOS |
| 8/9/19 | 60 | 6 | 7 | 5.948 | 13 | Low | Cool | MLOS |
| 8/9/19 | 60 | 7 | 7 | 6.195 | 13 | Low | Cool | MLOS |
